# Progress of laser surface treatment on magnesium alloy

**DOI:** 10.3389/fchem.2022.999630

**Published:** 2022-09-23

**Authors:** Shiliang Zhang, Jing Jiang, Xianrui Zou, Ning Liu, Hongshui Wang, Lei Yang, Huan Zhou, Chunyong Liang

**Affiliations:** ^1^ School of Materials Science and Engineering, Tianjin Key Laboratory of Materials Laminating Fabrication and Interface Control Technology, Hebei University of Technology, Tianjin, China; ^2^ Center for Health Sciences and Engineering, Hebei Key Laboratory of Biomaterials and Smart Theranostics, School of Health Sciences and Biomedical Engineering, Hebei University of Technology, Tianjin, China; ^3^ Changzhou Blon Minimally Invasive Medical Devices Technology Co., Ltd., Jiangsu, China

**Keywords:** magnesium alloy, laser treatment, mechanical property, wettability, corrosion property

## Abstract

Magnesium (Mg) metals have been widely used in various fields as one of the most promising lightweight structural materials. However, the low corrosion resistance and poor mechanical properties restrict its applications. Surface treatments are common approach to enhance the mechanical strength and corrosion resistance of Mg metals. Among them, laser surface treatment generates novel tissues and structures *in situ* on the sample surface, thereby improving properties of mechanical strength and corrosion resistance. We briefly describe the changes in surface organization that arise after laser treatment of Mg surfaces, as well as the creation of structures such as streaks, particles, holes, craters, etc., and provide an overview of the reasons for the alterations. The effect of laser processing on wettability, hardness, friction wear, degradation, biocompatibility and mechanical properties were reviewed. At last, the limitations and development trend of laser treatment on Mg metals research were further pointed out.

## 1 Introduction

Since the first discovery in 1808, Magnesium (Mg) metals have undergone two centuries of development and application. It takes an important role in the fields of automotive (Kainer, 2003; Luo, 2013; Gupta and Wong, 2015), aerospace (Mordike and Ebert, 2001; Huang et al., 2009), 3C products (Lorimer and Robson, 2008; Alaneme and Okotete, 2017) and biomedical materials (Zhai et al., 2014; Chen et al., 2015; Wagener et al., 2016; Li et al., 2018; Zoroddu et al., 2019). However, the low mechanical strength and corrosion resistance of Mg limit its development (Wang et al., 2014). Researchers have noticed that the mechanical strength and corrosion behavior of Mg metals are decided by the physicochemical properties, such as surface composition, structure, and roughness (Pardo et al., 2008; Lee et al., 2016). Therefore, appropriate surface modifications are needed to decelerate the corrosion rate and raise the mechanical strength and corrosion resistance of Mg metal.

**FIGURE 1:**
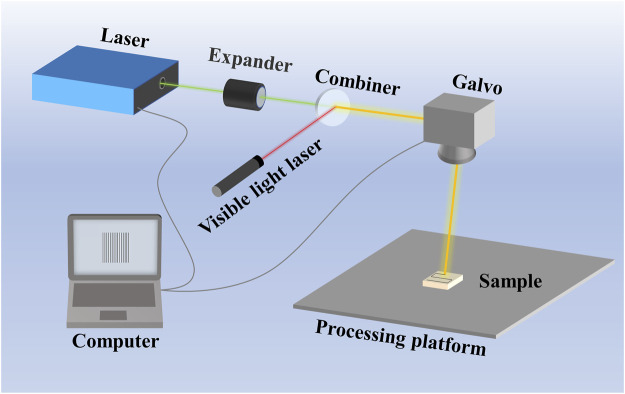
The schematic of laser surface treatment.

In recent years, researchers have been working on different surface modification measures used for Mg metals, including chemical conversion treatment (Huo et al., 2004; Zhang et al., 2021a), anodic oxidation treatment (Xue et al., 2011; Jothi et al., 2019), micro-arc oxidation (MAO) (Li et al., 2019c; Wang et al., 2019a; Zhang et al., 2019b), laser surface treatment (Lee et al., 2019; Park et al., 2021; Pou-Alvarez et al., 2021; Fajardo et al., 2022), chemical plating (Correa et al., 2013; Liu et al., 2020b) and electroplating (Wu et al., 2010; Arrabal et al., 2022), etc., Among them, laser surface treatment has the characteristics of high heat source power density and small heat input, which can realize the rapid heating and cooling of surface without thermal deformation (Majumdar et al., 2003; Liang et al., 2013; Wang et al., 2019b; Wu et al., 2020). As technology advances, laser beams can be precisely controlled by a computer to achieve accurate processing of any local position on the workpiece surface. When the laser beam irradiates a metal surface, the electrons on the surface are rapidly heating, rising the surface temperature of the irradiated and melting the metal surface. Moreover, due to the narrow range, the laser action region cools quickly after melting, which changes the structure and morphology of surface. The modulation of Mg alloy surface affects the metallurgy, mechanics, and physical properties, improving the corrosion resistance, wear resistance and biocompatibility (Pacha-Olivenza et al., 2020; Pulido-González et al., 2020; Zhang et al., 2021b; Fajardo et al., 2022). Figure 1 schematically shows an experimental setup used for laser direct writing in a sample, which consists of a laser, an expander, a combiner, a visible light laser, a galvo and a computer. The laser beam is passed through an expander for collimated laser to improve the focus afterwards to ensuring a high beam quality. Combined beam mirror is used to combine light and visible light for visualizing the laser. The sample was placed on the process stage, and the laser beam is focused on the sample surface and the processing path is designed through direct computer control of the galvo and the laser.

In this paper, we summarized the latest progress on laser treatment technology of Mg metals surface and analyzed problems and solutions. It is expected to provide ideas and references to solve the development and application of laser surface treatment technology of Mg metals.

## 2 Effect of laser surface treatment on organization and structure

### 2.1 Effect of laser surface treatment on microstructure characteristics

**FIGURE 2:**
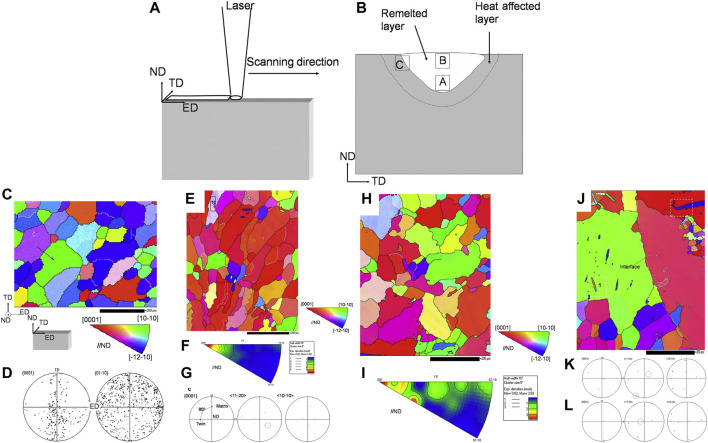
**(A)** The schematic of laser surface treatment and **(B)** selected areas on the cross section analyzed by EBSD; **(C)** a typical EBSD orientation map and **(D)** {0001} and {10–10} pole images of the initial sample; **(E)** EBSD OIM map of the bottom of laser melted layer, **(F)** the corresponding inverse pole figure, and **(G)** {0001}, <11–20> and <10–10 > stereographic pole figures corresponding to area one in **(E)**; **(H)** EBSD OIM map of the top of laser melted layer and **(I)** the corresponding inverse pole figure; **(J)** EBSD OIM map taken at the melt/substrate interface and the {0001}, <11–20>and <10–10 > stereographic pole figures corresponding to area 1 **(K)** and area 2 **(L)** in **(J)** (Zhang et al., 2014a).

The quick melting and cooling by laser irradiation will affect the surface organization and structure of the Mg metals surface. It is essential to understand the influence of different processes and environments on surface organizations and structures of Mg metals. Zhang et al. investigated the microstructure and grain growth behavior in the molten layer of pure Mg after CO_2_ laser surface treatment (Zhang et al., 2014a). The graded microstructure and texture layer was formed in the surface layer due to the rapid solidification and cooling after melting, as revealed in Figure 2. At the bottom of melted layer, Mg grains after solidified showed <0001> base fiber texture. While an almost equal number of particles are detected in top melted layer, the underlying fiber structure is much weaker. Solid and deformed twinning was found near the melt/substrate interface.

**FIGURE 3:**
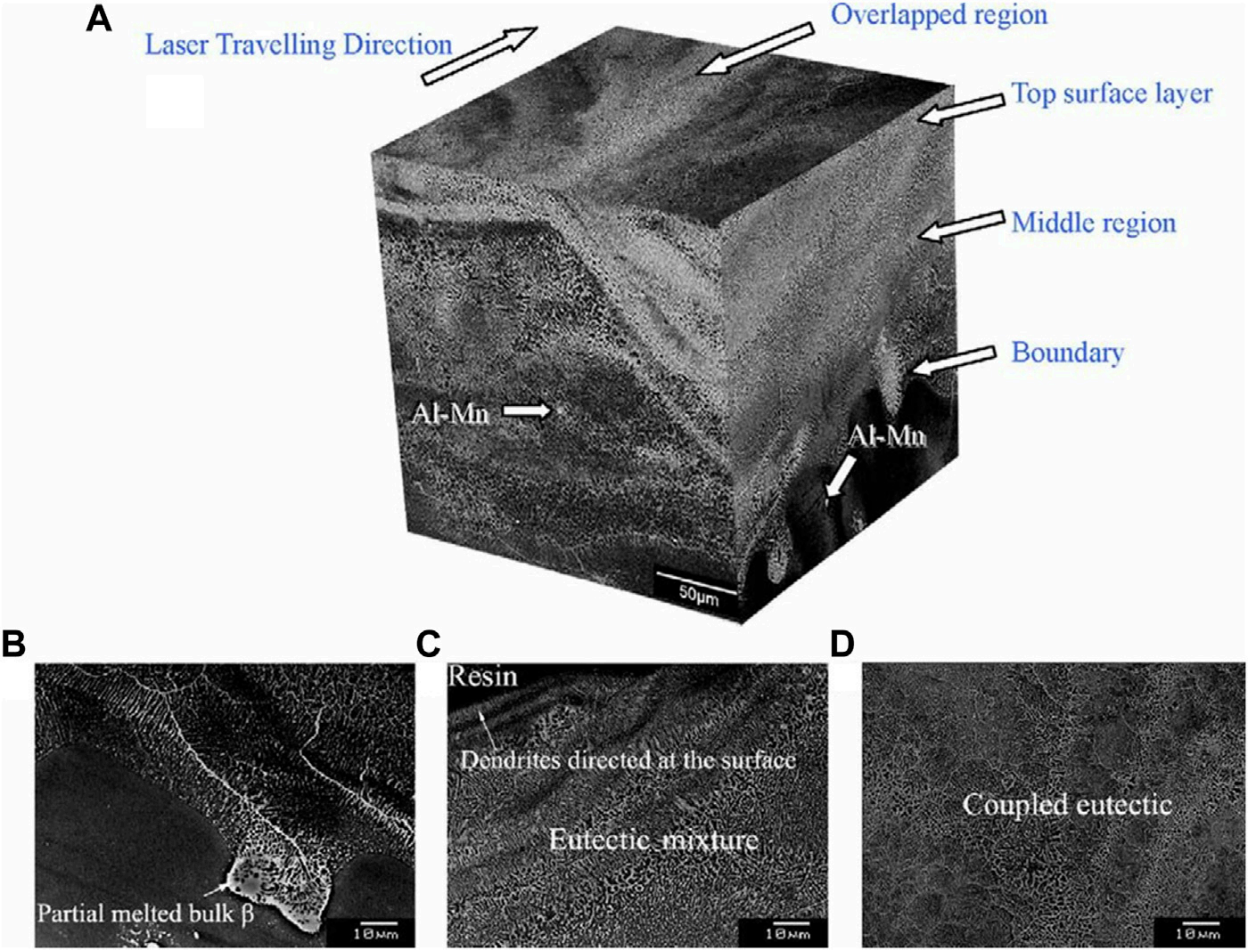
Images of typical solidification microstructure of AZ91D alloy after laser irradiation: **(A)** three-dimensional image of the laser-melted zone; **(B)** the boundary between laser-melted region and substrate; **(C)** the middle region; **(D)** the top surface. (Guan et al., 2010).

**FIGURE 4:**
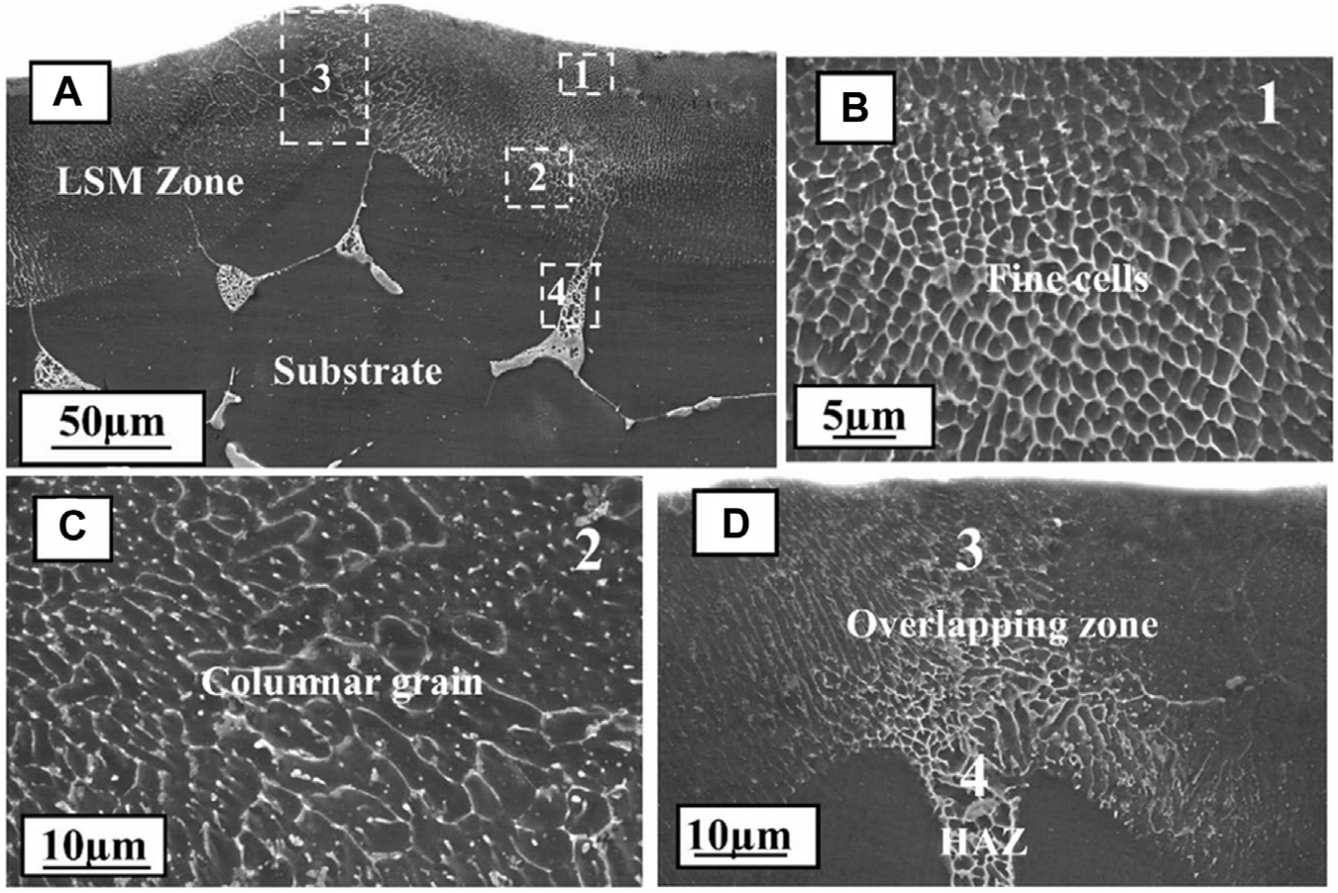
Cross-sectional SEM morphology of LSM samples processed: **(A)** microstructure of the melt pool (Rakesh et al., 2020).

Mg-Al series alloys are widely applied in a variety of fields owing to their excellent mechanical properties and have been extensively studied by academics. Using the Nd: YAG laser system, many scholars have investigated the influence of rapid solidification after laser melting on the structural evolution of AZ91 alloy (Guan et al., 2013a), AZ91D (Guan et al., 2009a; Guan et al., 2010), and AZ31B (Wu et al., 2017) alloys. Results demonstrated that, in the laser-melted region, the solidified microstructure is mainly composed of α-Mg cell/dendritic phase and a continuous network of β-Mg_17_Al_12_ (Taltavull et al., 2013a). AZ91D alloy treated by a high-power diode laser, the melt laser surface was obtained at high input laser energy, while low energy induces only one phase modified on the selective lase surface (Figure 3). The surface of AZ91 alloy (Iwaszko and Strzelecka, 2016) was treated by a continuous wave CO_2_ laser, in which the results showed that the remelted surface has a strong degree of refinement, and the phase distribution dominated by fine alpha-phase dendrites was homogeneous. The laser surface melting (LSM) of AM60B (Liu et al., 2015) and WE43 (Liu et al., 2016a) alloy by a continuous wave CO_2_ laser similarly, results revealed that the LSM-treated Mg alloys had uniform fine grains, abundant alloying elements and intermetallic compounds. The microstructure of the Mg-Zn-Gd-Nd alloy (Figure 4) treated by fiber laser surface modification is likewise consistent with the preceding results (Rakesh et al., 2020). In conclusion, the remelting of Mg alloy after the laser surface treatment result in an intense refinement of the surface organization and a high homogeneous phase distribution, detailed comparisons for various Mg alloys have been made in Table 1.

**TABLE 1:** Effect of laser surface treatment on the microstructure of Mg alloys.

	Substrate	After laser treatment
AZ91D Guan et al. (2009b)	Bulk and lamellar β-Mg_17_Al_12_ phase distributed non-homogeneously in a matrix of α-Mg grains	Solidification microstructure with a high degree of homogenisation and refinement and presented a continuous network of β-Mg_17_Al_12_ phase precipitated at dendrite/cellular boundaries and the primary α-Mg solid phase in the melted zone. The dendrite cell size was observed to increase with the decrease in cooling rates from the top surface to the bottom in the melted zone
Guan et al. (2010)		
Coy et al. (2010)		
Taltavull et al. (2013b)		
Guan et al. (2013b)		
ZE41 Khalfaoui et al. (2010)	An α-solid solution and intermetallic particles along the grain boundaries	α–Mg and Mg_7_Zn_3_(RE) phases same as untreated sample, but the intermetallic particles reprecipitated along the grain boundaries of the refined structure throughout the laser-treated zone, forming a refined and continuous network of precipitates
AM60B Liu et al. (2015)	α-Mg and eutectic mixture of α and β, as well as Al_8_Mn_5_ phases	The grain refinement, the Al element enrichment and the intermetallic compounds redistribution
AZ91 Iwaszko and Strzelecka, (2016)	The presence of large α-Mg grains and α+β eutectics (β—Mg_17_Al_12_ intermetallic compound) in the alloy structure, and the presence of secondary precipitations of β phase	Lead to a strong refinement of structure and a more even distribution of individual phases, and very fine α phase dendrites are formed in the structure of the surface layer, which are accompanied by β phase located in the interdendritic spaces.
WE43 Liu et al. (2016b)	α-Mg matrix and Mg_14_Nd_2_Y phase, as well as small amounts of Y-rich and Zr-rich phases	In modified layer, grains were refined, alloying elements were enriched in α-Mg matrix and intermetallic compounds were dissolved and redistributed
AZ31B Wu et al. (2017)	α-Mg	Equiaxed refined grains of α-Mg surrounded by a continuous network of β-phase (Mg_17_Al_12_) precipitated along the grain boundaries within the laser-melted region
Mg-Zn-Gd-Nd Rakesh et al. (2020)	Large equiaxed dendritic α grains with secondary phases (Mg_12_Nd) that surrounds the α-phase in the semicontinuous network.	The large equiaxed grains at or near the surface transformed to a fine grain network in the laser melted regions. And a transition occurs in the grain morphology through the depth of the melt pool from equiaxed to columnar.

The mechanisms that changing the microstructure of Mg alloys by different laser treatment are different. For the laser remelting treatment technology, surface layer is rapidly melting in the laser irradiation, and then natural cooldown. The melt pool emergences during the surface remelting process, the melting effects varies with the depth from the surface layer, so that the Mg alloy melt microstructure show gradient changes. The laser shock processing can also produce a gradient microstructure, which is due to the gradient characteristics of the laser impact blasting process, the grain size decreases with the increase of distance from the surface (Montross et al., 2002). When the shock wave propagates into the target, the intensity gradually decreases, which leads to a decrease in strain and strain rate, resulting in strain-induced microstructural changes (Hu et al., 2006). There are many kinds of laser treatment methods for Mg alloy, for example, laser remelting (Xiao et al., 2022), laser shock processing (Zhang et al., 2010; Zhang et al., 2020b; Liu et al., 2020a), laser alloying (Chen et al., 2010; Majumdar and Manna, 2010), laser cladding (Li et al., 2020; Chen et al., 2016). Among them, laser remelting and laser shock improve the Mg alloys properties by laser action without metallic attachments. While laser alloying and laser cladding both add components on the surface to fabricate a laser alloyed layer. For different laser surface treatment techniques, there are many factors affect the microstructure evolution, mainly the type of laser, the wavelength of the laser, the processing power, the processing speed, the processing environment, etc., (Singh and Habimkak, 2012; Liu et al., 2017; Cao et al., 2006).

### 2.2 Effect of the structure after laser surface treatment

**FIGURE 5:**
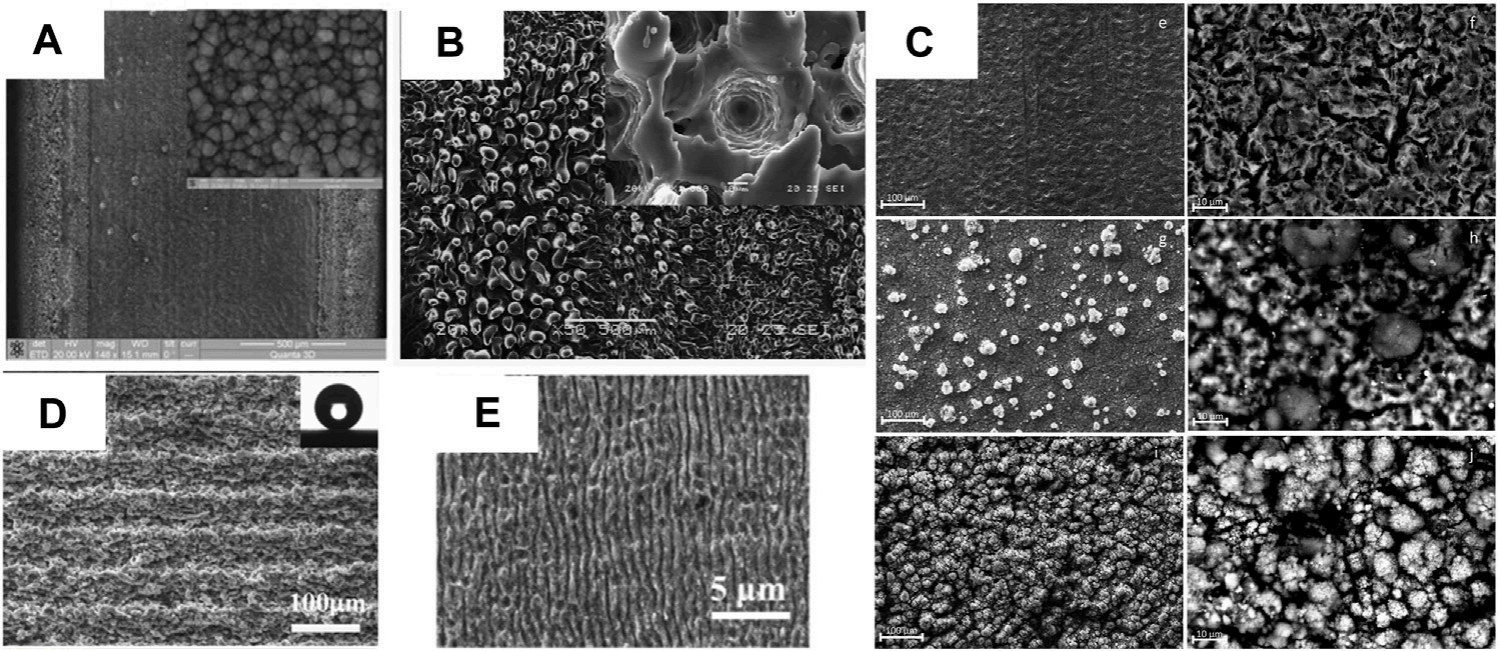
Surface morphologies. **(A)** AZ31B alloy surface after irradiation with progressive laser power of 110 W and high magnification images from the areas (Guan et al., 2013b); **(B)** Laser-induced craters by nanosecond pulsed Nd: YAG laser irradiation (Guan et al., 2014a); **(C)** the laser structure of AZ31 (Demir et al., 2014); **(D)** superhydrophobic AZ91 (Wei et al., 2019); **(E)** laser melted and LIPSS (Zhang et al., 2019a).

Researches on the impact of laser surface treatment on Mg metals have focused on the association between the process and the structure changing, intending to upgrade surface character of Mg metals by producing an ideal surface structure. Scholars have constructed different structures on the surface of Mg metals by various processing methods. Guan et al. utilized a nanosecond pulsed laser to treat the surface of Mg alloy, resulting in consistent projections at the treated groove places (Guan et al., 2013b). Guan et al. (2014a) also prepared micron craters on the Mg alloy by a nanosecond pulsed laser. Demir et al. (2014) employed a pulsed fiber laser to prepare distinct structures on an AZ31 surface and investigated the interaction between procedure and surface structure. Other researchers have used laser therapy to create various surface structures (Zhang et al., 2019a; Wei et al., 2019). Structures are shown in Figure 5.

**FIGURE 6:**
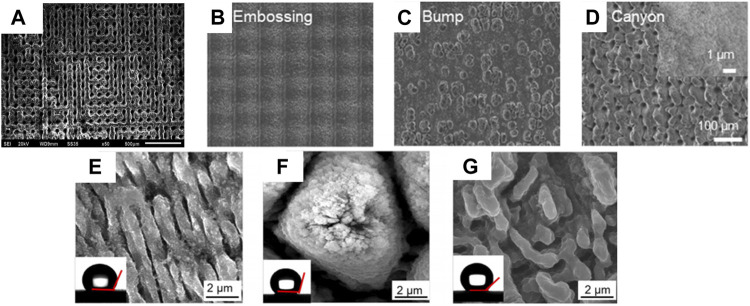
SEM images of laser textured surface from **(A)** (Aulakh and Kushal, 2019), **(B–D)** (Park et al., 2018), and **(E–G)** (Zhang et al., 2021b).

To improve the bonding of the coating, some scholars have performed laser surface treatments by Q-switch Nd:YAG laser system (Aulakh and Kaushal, 2019) and ytterbium femtosecond laser (Park et al., 2018; Zhang et al., 2021b) on the surface of Mg alloy, and structures are shown in Figure 6.

**FIGURE 7:**
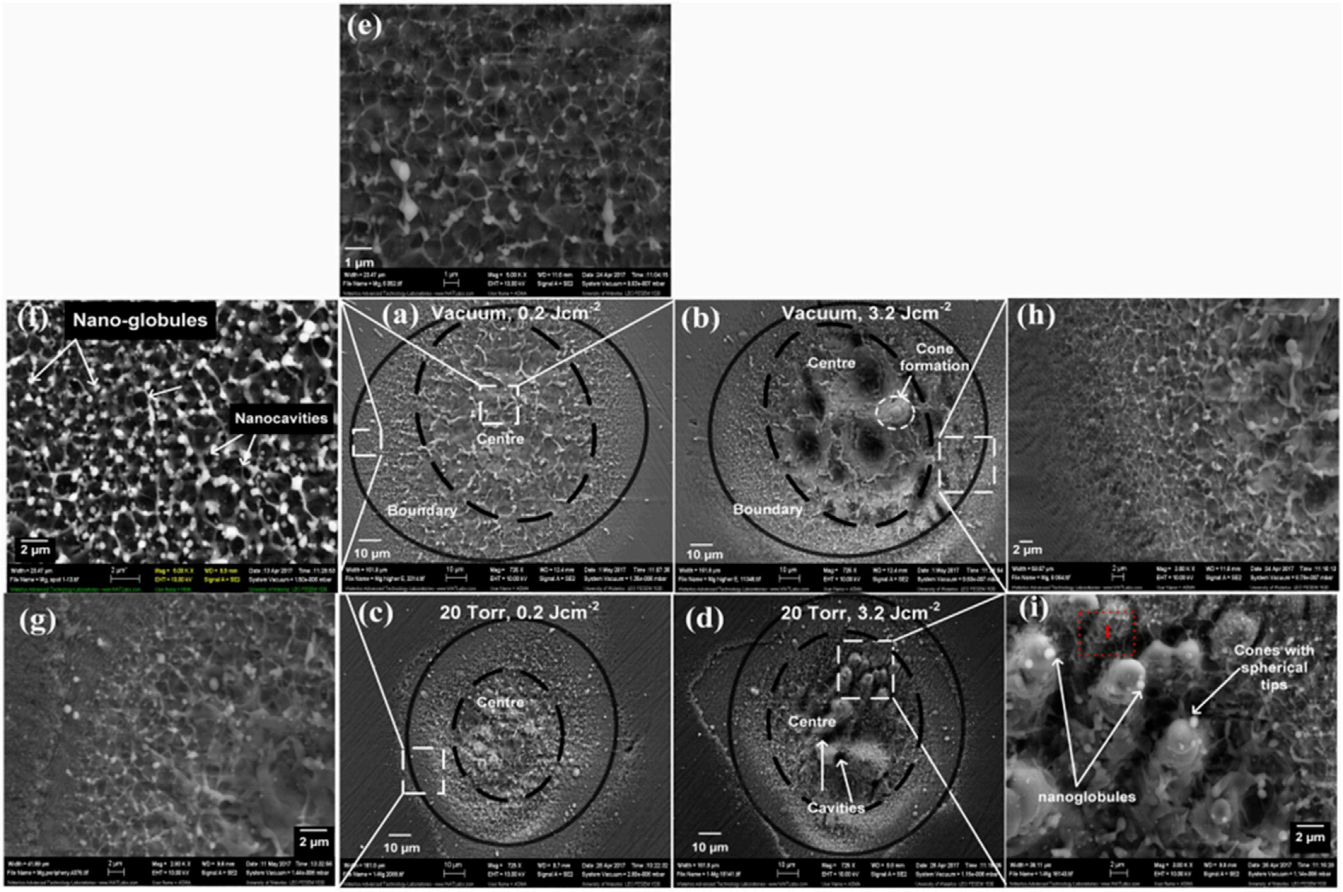
SEM micrographs revealing the surface morphology of the ablated regions of Mg under vacuum at different fluence: **(A)** 0.2 J/cm^2^ and **(B)** 3.2 J/cm^2^ and under an Ar pressure of 20 Torr at a fluence of **(C)** 0.2 J/cm^2^ **(D)** 3.2 J/cm^2^, **(A–D)** overall ablated area, **(E,G,H)** magnified peripheral ablated areas **(F)** and **(I)** magnified central ablated areas. (Hayat et al., 2019).

**FIGURE 8:**
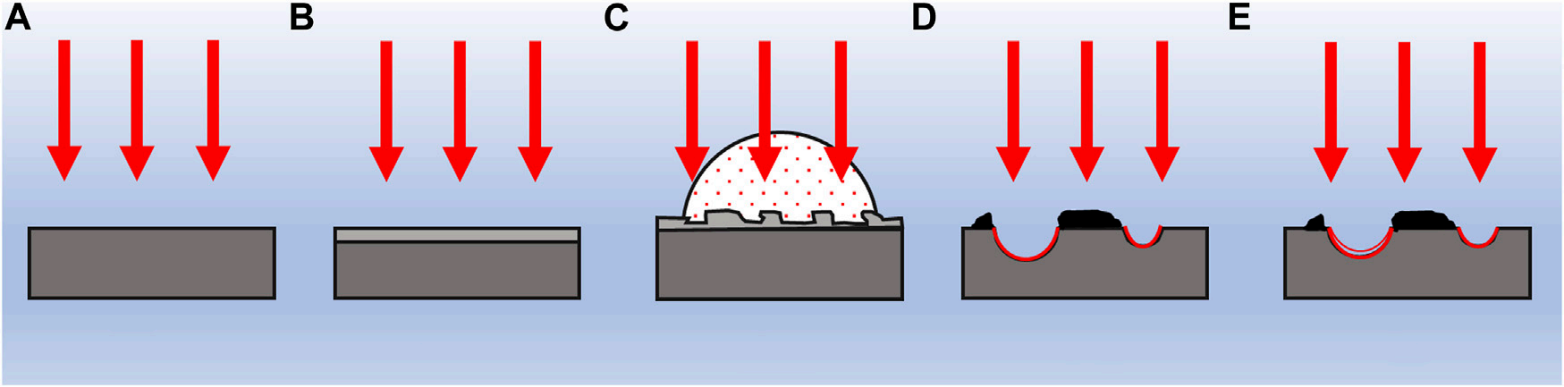
Schematic representation showing the stages of laser-induced crater formation: **(A)** surface absorption and thermal conduction; **(B)** surface melting (boiling); **(C)** plasma expansion and shock waves; **(D)** cavitation bubble generation; and **(E)** bubble oscillation and crater formation (Guan et al., 2014a).

Since the external environment affects the laser processing and changs the surface structure of metals, Hayat et al. (2019) used a Ti: Sapphire laser to study the impact of laser energy density, vacuum, and argon on the surface structure of Mg. In Figure 7, the surface of Mg metals irradiated under vacuum showed inhomogeneous nanospheres, nanocores, and micrometer structures, whereas the surface of Mg irradiated in Ar showed inhomogeneous micrometer cones covered by nanospheres. It has been suggested (Guan et al., 2014a) and explained that the micron-scale crater morphology produced by nanosecond pulsed laser irradiation of AZ91D alloy is mainly caused by laser-induced local boiling. The formation mechanism of metal surfaces is shown in Figure 8. The nanosecond pulsed laser irradiates the material surface and heating, causing the material to melt and creating a superheated sub-stable liquid layer on the surface, illustrated in Figures 8A,B. Motivated by the pressure and temperature difference above the surface of irradiation, the plasma is generated and expands, producing a high pressure shock wave on the surface, as depicted in Figure 8C. The highly superheated layer leads to mixed vapor-liquid droplets, which result in explosive boiling and bubble expansion. Nucleation of the gas phase occurs near the surface, leading to these “micro-explosions” inside the target during the incident laser pulse, causing volume expulsion of the material. Although the generation of some laser surface morphologies has been explained, however, there is a lack of in-depth research on the causes of other morphologies produced by laser processing.

## 3 Effect of laser treatment on the surface properties of Mg metals

### 3.1 Wear resistance

Wear resistance is used to characterize the ability of a material to resist wear during friction. Wear-resistant materials require low friction factor, sufficient strength, strong compressive stress, and shear resistance, as well as certain toughness, oxidation resistance, impact resistance, fatigue resistance, high temperatures resistance, good thermal conductivity, and good wettability. In general, wear-resistant materials have good comprehensive performance and excellent wear resistance, surface treatment is a significant method to improve the wear resistance of the material. Changes in surface organization and structure are demonstrated to be the reason for improving wear resistance of Mg metals. The laser surface treatment result in a high uniform (Yao et al., 2008), fine (Li et al., 2010), and hard (Zhang et al., 2014b) surface organization, all of these led to an increase in the wear resistance of Mg metals. Taltavull et al. (2013b) used a high-power diode laser (HPDL) for surface treatment of AM60B alloy, investigating the surface wear resistance and presenting the wear mechanism of laser treated AM60B alloy, and wear tests were carried out under dry sliding condition on a pin-on-disc tribometre, and the wear test parameters were selected based on the sliding wear mechanism map of the AM60B. At the applied load of 10 N and the sliding speed of 0.1 m s^−1^, abrasion and oxidation were the dominant wear mechanisms as observed. After increase in the applied load (from 10 to 150 N) and sliding speed (0.1–0.3 m s^−1^), delamination and oxidation were identified as the dominant wear mechanisms. Continue increasing the sliding speed from 0.5 to 1 m s^−1^ at 150–250 N, leading to plastic deformation. Many other scholars have studied impact of laser surface modification on wear resistance of Mg metals with different lasers and process parameters, and results showed the wear resistance of laser-treated Mg alloys was successfully improved (Zhang et al., 2008; Iwaszko and Strzelecka, 2016; Zhou et al., 2016; Zhang et al., 2019a; Zeng et al., 2019).

### 3.2 Mechanical property

#### 3.2.1 Hardness

Hardness is an important performance index to measure the softness and hardness of metals, indicating the ability of a material to resist damage. Surface treatment is an effective approach to higher the hardness of materials, and surface of Mg alloy after laser surface treatment has different organization and structure, changing its hardness. Studies have shown that surface hardness of Mg metals increased mainly due to the grain refinement of the metal structure after laser surface modification (Khalfaoui et al., 2010; Iwaszko and Strzelecka, 2016; Pulido-González et al., 2020; Rakesh et al., 2020). Nevertheless, since the existence of the separated second phase (Li et al., 2019a), fluctuating changes in hardness values may be caused. The variation of surface hardness of laser modified Mg metals is shown in Table 2.

**TABLE 2:** The variation of hardness on the surface of laser-modified Mg alloys.

	Substrate	Melt layer	Enhancement	Laser system	Process parameters
MEZ Majumdar et al. (2003)	35 HV	100 HV	2.86	a continuous wave CO_2_ laser	Q = 1.5 kW, V = 200 mm/min
ZE41 Khalfaoui et al. (2010)	72 HV	124 HV	1.72	a Lambda Physik excimer laser	E_p_ = 100 mJ, V = 50 μm/s
AZ91D Taltavull et al. (2013a)	69.1 HV	107.5 HV	1.56	a continuous wave high-power diode laser	Q = 600 W V = 90 mm/s
AZ91 Iwaszko and Strzelecka, (2016)	60 HV	93 HV	1.55	A continuous wave CO_2_ laser	Q = 900W, V = 33.3 mm/s
MB26 Li et al. (2019a)	70 HV	105 HV	1.5	nanosecond pulsed fiber laser	*p* = 1.20 × 10^7^ W/cm^2^
AZ80 Li et al. (2019a)	80 HV	100 HV	1.25		V = 200 mm/s
AZ91D Meng et al. (2017)	60 HV	112 HV	1.87	A solid state Nd^3+^: YAG laser	E_p_ = 18
					J, v = 1 mm/s
Mg-1Zn-1Ca Pulido-González et al. (2020)	61.7 HV	76.4 HV	1.23	A High Power Diode Laser	Q = 743 W, V = 85 mm/s
Mg-3Zn-0.4Ca Pulido-González et al. (2020)	60.7 HV	77.3 HV	1.27	A High Power Diode Laser	
AZ31B Xu et al. (2018)	55 HV	109 HV	1.98	The ultrasonic vibration-assisted laser surface melting system	Q = 600 w V = 600 mm/min

#### 3.2.2 Strength

As the lightest structural metal, Mg metals have been important materials for automotive and aerospace applications for many years, therefore, enhancing the mechanical properties of Mg metals has been the focus of attention in various fields. One of the most widely used commercial Mg alloys is the Mg-Al series, the microstructure of a typical Mg-Al binary system consists of two main phases: α-Mg matrix and β-Mg_17_Al_12_. The mechanical properties were reported to be adversely impacted by existence of the brittle β-Mg_17_Al_12_ phase (Zhang et al., 2015). Furthermore, some researchers believe that laser surface treatment is an attractive method to promote the mechanical behavior of Mg metals.

Taltavull et al. (2014a) performed three-point bending tests by *in situ* scanning electron microscopy to monitor the fracture process of SLSM-treated AZ91D alloy, results showed that microstructural of brittle β-Mg_17_Al_12_ phase modification to continuous network phase by SLSM treatment to prevent brittle fracture, the fracture toughness was increased by 40.3% compared with the AZ91D alloy.

Zhang et al. (2015) treated AZ91D alloy surface with pulsed Nd: YAG laser, reducing the number of irregular β-Mg_17_Al_12_ and crack nucleation sites, improving the thermal fatigue resistance of the material. Meng et al. (2017) treated the surface of Mg alloy with a laser and performed tensile tests at room temperature and high temperature. Results detected that the strength of LSM-treated samples improved and the ductility decreased compared to the reference samples. It is mainly due to the non-dynamic recrystallization of the laser melting zone deflecting the fracture cracks and affecting the stress transfer, leading to an increase in the strength of the LSM-treated samples at high temperatures. Meng et al. (2018) also investigated the influence of laser treatment on fracture behavior of Mg alloys at different temperatures and strain rates, and results showed that the mechanical properties of the LSM-treated samples were better than those of the original samples.

In summary, laser surface treatment of Mg metals mainly changes the organization and structure through the rapid heating and cooling effects, thereby changing properties of Mg metal. After laser irradiation of the Mg alloy surface, rapid melting and cooling of the action area occurs, resulting in a recrystallization process. The intermetallic phases show grain refinement and uniform distribution, which improves the mechanical properties of the material. By laser surface treatment, the property of wettability, hardness, wear resistance, and biocompatibility of Mg metals can be improved, and the mechanical properties also can be affected. However, studies on the influence of laser-treated surfaces on the mechanical properties of Mg metals in complex environments are not yet widespread.

### 3.3 Wettability

The wettability of the solid surface is determined by the chemical composition and microstructure. The higher free energy of the solid surface, the easier to be wetted by the liquid. Therefore, the searching and preparing of solid surfaces with high surface free energy or low surface free energy become a prerequisite for the preparation of superhydrophilic and superhydrophobic. Scholars (Demir et al., 2014; Rakesh et al., 2019; Rakesh et al., 2020) used laser surface treatment to change the surface organization or structure of Mg metals, to achieve the purpose of changing the surface energy for control the wettability.

Rakesh et al. (2018) performed laser treatment on Mg-1Zn-2Gd alloy and revealed that the changes in surface geometry, grain size, and surface roughness of LSM samples affected their surface energy and hydrophilicity. After low energy density laser irradiation, grain refinement, and surface roughness reduction occurred on the metal surface, resulting a better wettability of the sample surface. Bhaskar et al. treated Mg-2.2Zn alloy using the same method and obtained the same results (Manne et al., 2018). To obtain a surface with extremely high surface energy, Wei et al. (2019) prepared superhydrophilic and superhydrophobic surfaces on AZ91 alloy by a combination of laser ablation and annealing, and the laser ablated superhydrophilic surface was transitioned to superhydrophobic with a water contact angle up to 158.8 ± 2° after annealing at 160°C for 60 min. The shift of wettability is mainly attributed to microstructure and absorption of surface hydrophobic organics.

Most changes in the wettability of laser-treated Mg metal surfaces are attributed to the combined effects of the surface compound, the organization, and microstructure after laser irradiation. For Mg metals, the change of wettability affects the degradability of Mg metals, however, the mechanism of the combined effect of wettability as well as surface structure on the degradation behavior has not been comprehensively investigated.

### 3.4 Corrosion property

The standard electrode potential of Mg is −2.37 V (SCE), So it is chemically active and vulnerable to erosion damage in acidic, neutral and weak alkaline media, hence the corrosion behavior of Mg metals has been the focus (Chen et al., 2015; Liu et al., 2019; Jiang et al., 2020). The corrosion behavior of Mg metals has two categories, uniform corrosion and localized corrosion. The corrosion behavior of Mg alloy can be modified by laser surface treatment which precisely controls the surface treatment region. However, effects of different surface treatment procedures on the corrosion behavior of Mg metals are inconsistent.

**FIGURE 9:**
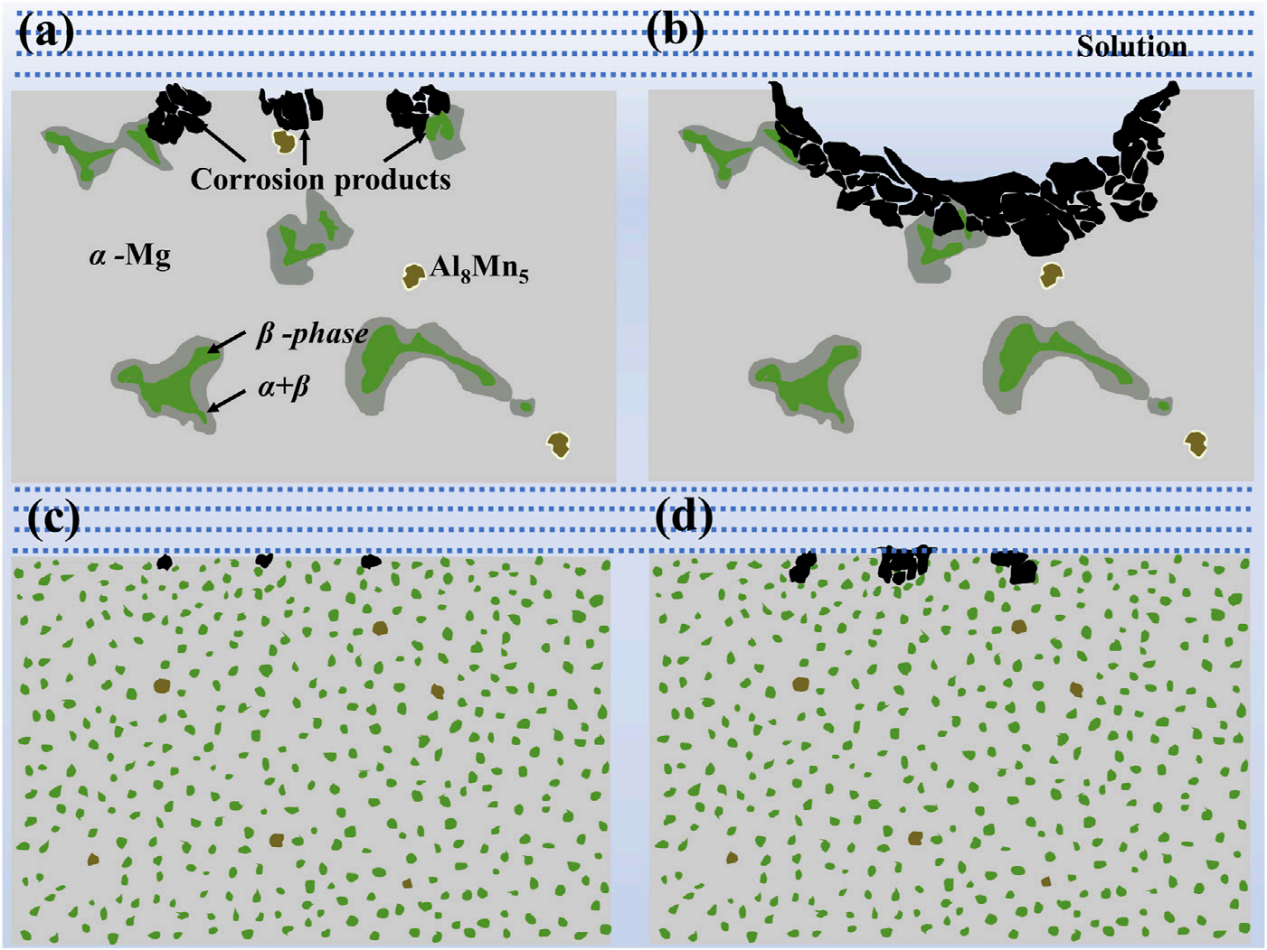
Schematic diagrams for the corrosion process of the as-received **(A,B)** and LSM treated **(C,D)** AM60B alloy (Liu et al., 2015).

The previous report on laser treatment of Mg alloys to retard degradation came from Abbas et al. (2005) where LSM of Mg alloys were performed using a 2 kW continuous wave CO_2_ laser. Results showed that the weight loss of AZ31, AZ61, and WE43 alloys was reduced by about 30%, 66% and 87%, respectively, after laser surface treatment. The analysis showed that rapid cooling of the alloy melt layer after LSM led to the refinement of microstructure, the refined microstructure promoted uniform corrosion. After the surface treatment, the β-phase distribution was homogeneous, easy to accumulate and form a barrier layer. A large number of scholars subsequently performed different laser treatments on Mg metals to improve their corrosion resistance, the results of corrosion rate are shown in Table 3. Among them, Liu et al. (2015) used a 10 kW continuous-wave CO_2_ laser to perform the surface melting of AM60B Mg alloy to increase the corrosion resistance of AM60B alloy. The improvement of corrosion resistance was mainly due to the reduced corrosion susceptibility of the Al-Mg-rich matrix, and the potential barrier effect formed by the homogeneous β-phase distribution (Figure 9).

**TABLE 3:** Comparison of corrosion rate in different solutions after laser treatment.

Materials	Substrate (A/cm^−2^)	Laser treatment (A/cm^−2^)	Solution	Laser	Process Parameters
AZ91D	7.22 × 10^−5^	7.50 × 10^−6^	SBF	a Lumonics JK704 Nd:YAG laser system Guan et al. (2009b)	E_p_ = 3.82 × 10^4^ W/cm^2^, V = 10 mm/s
	1.20 × 10^−6^	5.50 × 10^−7^	3.5 wt% NaCl	a Lumonics KrF pulsed-excimer laser Coy et al. (2010)	*p* = 6.0 J/cm^2^
AM60B	4.48 × 10^−6^	5.98 × 10^−7^	3.5 wt% NaCl	a continuous-wave CO_2_ laser Liu et al. (2015)	in Ar, V = 1000 mm/min *p* = 89 J/mm^2^.
WE43	2.93 × 10^−5^	8.51 × 10^−6^	0.62 M NaCl	a continuous-wave CO_2_ laser Liu et al. (2016a)	In Ar, Q = 3000W V = 1000 mm/min
AZ31B	2.17 × 10^−4^	1.10 × 10^−7^	3.5 wt% NaCl	a continuous wave fiber laser Xu et al. (2018)	Q = 600W V = 600 mm/min
	1.214 × 10^−5^	5.82 × 10^−6^	SBF	a continuous-wave 3-kW Nd:YAG laser Wu et al. (2017)	p = 3.18 J/mm^2^ V = 500 mm/s
Mg-Zn-Gd	8.0 × 10^−6^	1.3 × 10^−6^	Hanks	a continuous wave Ytterbium doped fiber laser Rakesh et al. (2018)	*p* = 0.535 J/cm^2^ V = 2 mm/s
Mg-Ca-Zn	1.97617 × 10^−5^	9.3619 × 10^−6^	Hank’s	a ytterbium fs laser (Park et al., 2018)	*p* = 5.2 J/mm^2;^ V = 200 mm/s
Mg-Gd-Ca	5.54 × 10^−5^	2.07 × 10^−6^	Hank’s	a Ti:sapphire chirped-pulse regenerative amplification laser system Zhang et al. (2019c)	E_p_ = 2.04 × 10^6^ W/cm^2^, V = 60 mm/s
MB26	5.6.8 × 10^−5^	3.12 × 10^−5^	3.5 wt% NaCl	a nanosecond pulsed fiber laser Li et al., (2019a)	E_p_ = 1.20 × 10^7^ W/cm^2^ V = 200 mm/s
AZ80	9.60 × 10^−5^	3.79 × 10^−5^	3.5 wt% NaCl	a nanosecond pulsed fiber laser (Li et al., 2019a)	E_p_ = 1.20 × 10^7^ W/cm^2^ V = 200 mm/s
Mg-10Li-3Al-3Zn	3.00 × 10^−5^	7.63 × 10^−7^	0.1 M NaCl	a Nd: YAG laser Zhang et al. (2020a)	Q = 3500 W V = 10 m/min

To achieve further improvement of the corrosion resistance of Mg alloys, some scholars have used alternating magnetic field assisted laser surface treatment (Zhou et al., 2016), grinding (Taltavull et al., 2014b) or annealing treatment (Wei et al., 2019), they all significantly improved the corrosion performance of Mg alloys.

Although studies have shown that the corrosion resistance of Mg metals could be improved by laser surface treatment, Coy et al. (2010) detected the corrosion performance of polymer LSM of die-cast AZ91D alloy and showed that increasing the number of laser pulses enhanced the porosity of the metal surface and formed microcracks in the overlapping areas, which reduces the corrosion resistance of the laser-treated alloy.

**FIGURE 10:**
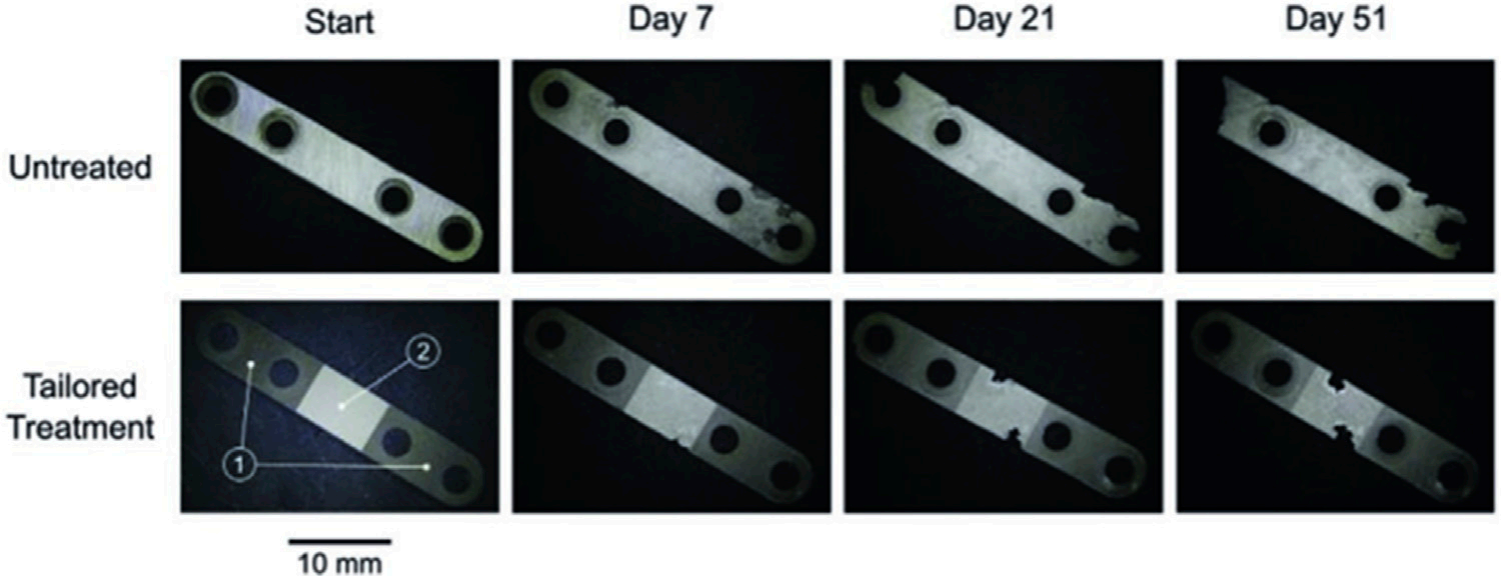
Macroscopic degradation performance of untreated and tailored-treated bone fracture fixation plates (Pou-Alvarez et al., 2021).

Corrosion is generally considered to be avoided, although applications like batteries and biodegradable implants would benefit. Some researchers (Pou-Alvarez et al., 2021) guide the corrosion process of Mg alloys through local control of laser surface treatment. Depending on the local control afforded by the laser treatment, corrosion can be restricted to the region of interest and driven in a specific direction by selectively adjusting the overall corrosion rate in each region. Results demonstrate the applicability of the method and provide a reference for the design of custom degraded implants to suit the different tissue requirements and environmental conditions in different areas of the implant (Figure 10).

The degradation properties of Mg metals can be influenced by building different surface structures. Microstructures of recast layer explains the corrosion changes of the treated samples. Mg alloys with laser recast layer possess a dense protective layer, which will improve the corrosion resistance. In contrast, a porous/broken laser layer on the sample surface accelerates the corrosion of Mg metals. The increase in corrosion resistance of laser-treated Mg alloys is mainly due to the hindrance of galvanic corrosion. The galvanic coupling corrosion of Mg alloys is caused by the adjacent α-phase and the potential difference of the intermetallic compound. While the β-phase in the alloy plays a dual role in corrosion, acting as a current cathode or barrier, depending on its size, distribution and fraction. After laser-treatment, the fine-grained intermetallic compound is uniformly distributed in the α-Mg matrix, which reduces the potential difference and alleviates galvanic corrosion. The uniformly distributed β-phase after laser treatment formed an almost continuous mesh in the corrosive environment, which further hindered the corrosion (Liu et al., 2015). Applicating different processes to guide and regulate the degradation, by precisely constructing different surface structures on the surface of Mg metals, opening the door for laser-guided corrosion control concepts to be applied to other areas where corrosion of controlled materials is needed.

### 3.5 Biocompatibility

Biocompatibility refers to the biological properties of medical implant materials that can withstand the action of various host systems, and maintain a relatively stable state without being rejected or destroyed during the dynamic changes in the organism. The biological reaction of human tissues to the implants usually occurs at the interface between the material and the contacting organism, including cell surface-extracellular matrix, cell surface-implant surface, etc. (Muskovich and Bettinger, 2012). Cells are the basic unit for sustaining life and the basic substance for tissue repair (Janes et al., 2002). Therefore, biocompatibility is not only dependent on the material itself but also closely related to the properties of the material surface in direct contact with human cells, including the structure, composition, surface morphology, hydrophobicity and energy state of surface (Curran et al., 2005). Improving the surface properties through biological, chemical and physical methods, significantly improves the biocompatibility of medical implants with living organisms without changing the *in situ* physical properties of material. From the perspective of materials science, the microstructure on the surface of biomaterials determines the properties, so the biocompatibility of implants can be improved by preparing microstructures on the surface to influence cellular behavior, which was previous reported by Harrison (1912). Since then, a large number of studies have a focused on using surface morphology to guide cell growth and development. In recent years, ultrafast lasers have successfully induced micro and nanostructures on the surface of medical biomaterials and devices to influence cellular behavior, thus improving their biocompatibility (Brunette et al., 2001).

Although Mg metals have received a lot of attention as degradable biomedical materials, not much research has been carried on the biocompatibility of Mg metals after laser surface treatment. This is mainly because although the laser layer can retard corrosion in the pre-corrosion stage, it can accelerate the degradation of Mg metals, after immersion in the solution for a period due to increasing the specific surface area. Lu et al. investigated the biomineralization behavior of laser treated samples in simulated body fluids with laser surface treatment using a continuous-wave Nd: YAG laser on AZ31B alloy (Lu et al., 2019). Results showed a significant improvement in biomineralization at an optimum laser fluence of 3.286 J/mm^2^. Zhang et al. proposed a hybrid laser surface modification method of laser melting and laser surface texturization on Mg-Gd-Ca alloy to study the adhesion and growth behavior of MC3T3-E1 cells *in vitro* (Zhang et al., 2019a). Results showed that the submicron surface structures produced by the femtosecond laser on the melting surface could provide durable mechanical stimulation to the cells, thus allowing controllable cell shape. The micron surface structure produced by the picosecond laser on the melting surface affects the cell distribution owing to the cell rejection effect. The effectiveness of the hybrid laser approach to improve the biocompatibility of Mg-Ca alloys.

Although the biological application of Mg Metals is a hot research topic in recent years, the research on the biocompatibility of Mg alloys is not comprehensive enough, there is a lack of study on the relationship between microstructure of surface, degradation behavior and biocompatibility, for example, the effect of changes in surface structure during degradation on cell adhesion, proliferation, and differentiation. Therefore, futuristic works can be conducted to investigate effect of degradation processes of Mg alloys with different surface structures on the cellular behavior, by constructing *in vitro* biological models.

### 3.6 Effect of surface absorbance

The laser induced light absorbance effect is an important phenomenon in Mg alloys offering potential engineering applications in product identification, photocatalysts and bio-optical implants (Guan et al., 2013b; Guan et al., 2014b; Guan et al., 2014c; Shi et al., 2015). The structure and composition of the laser-treated surface affect the light absorption of magnesium alloys, causing a color change in the metal surface.

**FIGURE 11:**
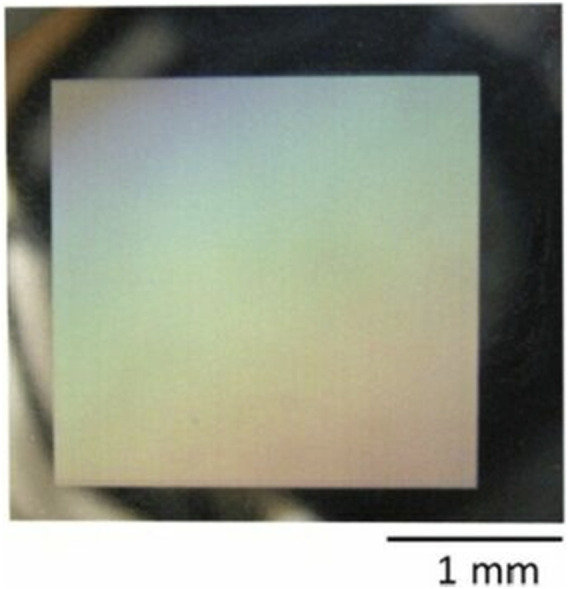
Image of typical iridescent effect on the irradiated surface by femtosecond laser (Guan et al., 2014b).

Guan et al. (2014b) reported the microwave ripples and nano ripples on the surface of AZ31B alloy irradiated by femtosecond laser and explored the femtosecond laser beam induced iridescence effect on a large radiating surface. The results indicated the color effect was mainly due to the extensive period distribution of the nano ripples as diffraction gratings, while the intensity of the structural color was strongly influenced by the morphological evolution of the microwave ripples after laser treatment. Subsequently, scholars have structured the surface of Mg alloy by using nanosecond, excimer, and femtosecond lasers, and the results all show that the darkening effect on the surface of Mg alloy after laser treatment is a joint effect of the laser-treated structure and the increased oxygen content in the laser-treated region (Guan et al., 2013b; Guan et al., 2014c; Shi et al., 2015). Due to the different absorbance rates of laser treatment surface structures, the absorption and reflection of light. By modulating the laser characteristics to the thermal properties of the material, the technique can be widely employed, providing potential for the development of new magnesium-based bio-optics and color displays. The image of typical iridescent effect on the irradiated surface was shown in Figure 11.

Although the simple laser surface improved mechanical properties and corrosion resistance, it is hard to meet all the needs of practical applications to achieve a single function. In recent years, a large number of scholars have shown great interest in building laser composite coatings.

## 4 Laser surface composite coating

To improve surface properties for the applications, it is often necessary to construct composite coatings on Mg metals (Patel et al., 2010). It is critical to select the right coating/substrate interface for taking advantage of coatings. The bond strength of the coating on the metal substrate is enhanced by creating micro-and nano-patterns changing the surface morphology of the Mg alloy. The surface patterns produced by laser treatment increase the surface area and enhance the mechanical engagement at the bonding interface (Zhang et al., 2021; Park et al., 2021). For degradable polymer biofilm layer, the surface energy of Mg alloys has a great influence on the bonding force of the film layer to the substrate. Laser treatment regulates the surface energy of Mg metal, controls the wetting state of polymer film layer solution and substrate surface, to achieve the purpose of controlling the film layer and substrate binding force.

To enhance the corrosion resistance of Mg alloys, some scholars (Chen and Thouas, 2015; Li et al., 2019b; Zhang et al., 2019) have performed LSM pretreatment and micro-arc oxidation (MAO) composite treatment on Mg alloys. The LSM pretreatment results in a thicker and more refined and homogeneous MAO coating. It is favorable to the formation of MAO coating, increases the bonding force and the corrosion resistance of Mg alloy.

Liu et al. (2016b) and Aulakh and Kaushal (2019) used laser texturing as a substrate preparation technique to prepare ceramic coatings by PEO and thermal spray surface treatment techniques, respectively, which not only increased the bonding strength of the coating, but also satisfactory improved the corrosion resistance.

Biodegradable polymer coatings are usual as barriers to further enhance the advantages of implantable materials, and also require pretreatment of metal surfaces to enhance the adhesion strength of polymers to the metal surfaces. Researchers (Ali et al., 2015; Zhang et al., 2021b; Park et al., 2021) have further improved the biocompatibility and modulated the degradation behavior of Mg alloys by surfaces laser treatment to enhance the bonding strength of polymer coating.

There have been many studies on the construction of composite coatings by laser performed coatings, which achieved their intended purpose. However, most of the studies have focused on the overall large area coating construction, lacking the combination with precise laser control. In the future, the construction of composite coatings at specified locations can be realized by precisely controlling the laser processing area to meet the practical application requirements of complex conditions.

## 5 Conclusion and overviews

In current researches, mechanistic studies on the effect of laser on magnesium alloys have been reported, but there are still limitations. The effect of laser processing on the surface organization and structure of Mg metals needs to be explored in depth. Secondly, the mechanical properties of laser treated Mg metals have been studied, but they are not comprehensive enough, especially in corrosive environments. Although there are many studies on the degradation behavior of Mg metals after laser surface treatment, the explanation of the reasons for the change in degradation behavior is not perfect and lacks mechanistic understanding. While as the most promising biodegradable metal, Mg metals is a hot spot for research, but the current researches on the biocompatibility of laser treated Mg are not comprehensive enough. There is a lack of research on the relationship between microstructure, degradation behavior and biocompatibility, for example, the effect of microstructure on biocompatibility during degradation. For future research directions of laser treatment of Mg metal surfaces, the following possibilities are conjectured:

1) To establish a model of the influence of laser treatment on the surface organization and structure, and to clarify the effect law of laser treatment on the evolution of the surface organization and structure.
2) In-depth study of the effect of laser surface treatment on the mechanical properties of Mg metal, especially the corrosion fatigue resistance behavior, under realistic experimental conditions.
3) Establish the three-dimensional controlled degradation model to study the degradation behavior of surface laser treatment modulation, and establishing a network of relationships between laser surface structure and degradation behavior of magnesium alloys. Thereby, applying the controllable degradation developed to three-dimensional devices to provide a reference for practical applications.
4) Construct the complete database for the relationship between surface morphology and surface properties produced by different lasers treatment, which facilitate the rapid retrieval and selection of engineering applications.
